# Hepatectomy for metachronous colorectal liver metastases following complete cytoreductive surgery and hyperthermic intraperitoneal chemotherapy for peritoneal metastases: a report of three cases

**DOI:** 10.1186/s12957-019-1646-0

**Published:** 2019-06-13

**Authors:** Kyoji Ito, Nobuyuki Takemura, Fuyuki Inagaki, Fuminori Mihara, Toshiaki Kurokawa, Yoshimasa Gohda, Tomomichi Kiyomatsu, Hideaki Yano, Norihiro Kokudo

**Affiliations:** 1 Hepato-Biliary-Pancreatic Surgery Division, Department of Surgery, National Center for Global Health and Medicine, 1-21-1 Toyama, Shinjuku-ku, Tokyo, 162-8655 Japan; 2 Department of Surgery, National Center for Global Health and Medicine, 1-21-1 Toyama, Shinjuku-ku, Tokyo, 162-8655 Japan

**Keywords:** Colorectal cancer, Multi-modality treatment, Peritoneal metastases, Cytoreductive surgery, Hyperthermic intraperitoneal chemotherapy, Liver metastases, Hepatectomy

## Abstract

**Background:**

Cytoreductive surgery (CRS) with hyperthermic intraperitoneal chemotherapy (HIPEC) for peritoneal metastasis (PM) from colorectal cancer (CRC) has been reported to substantially improve the prognosis and the quality of life of patients in comparison to systemic chemotherapy or palliative approaches. This study aimed to demonstrate the safety and feasibility of hepatectomy for metachronous liver metastases from CRC following CRS and HIPEC for PM on the basis of three case reports.

**Case presentation:**

We describe three cases involving patients who underwent hepatectomy for metachronous liver metastases from CRC after CRS and HIPEC for PM. All patients underwent CRS and HIPEC after primary tumor resection, and hepatectomy was performed for the metachronous liver metastases after CRS and HIPEC. The hepatectomy procedures for cases 1, 2, and 3 were left hemihepatectomy and partial resection of S5, posterior sectionectomy, and left-lateral sectionectomy and partial resection of S5 and S8, respectively. Although adhesion of surrounding organs to the liver surface was observed on a broad level, dissections and hepatectomy could be performed safely. No recurrence was detected in cases 1 and 2 after hepatectomy. In case 3, liver metastases were detected from the time of the initial diagnosis of the primary tumor, and complete remission was achieved once with systemic chemotherapy. Although we performed hepatectomy for the recurrence of liver metastases after complete remission, early re-recurrence was observed after hepatectomy.

**Conclusions:**

Hepatectomy for metachronous liver metastases after CRS and HIPEC for PM could be a multi-modality treatment option for CRC recurrence.

## Background

Distant metastases from colorectal cancer (CRC) to the peritoneum, i.e., peritoneal metastases (PM), develop in 10–25% of CRC patients [1, 2]. Historically, PM from CRC were regarded as a terminal condition. Treatment was mainly based on palliative chemotherapy with poor long-term survival [2, 3], although some authors have reported a relatively favorable prognosis after simultaneous resection of PM and primary CRC in selected patients [4, 5].

Cytoreductive surgery (CRS) with hyperthermic intraperitoneal chemotherapy (HIPEC) for PM in patients with CRC has been shown to substantially improve the prognosis and quality of life compared with systemic chemotherapy or palliative approaches [6–12]. However, up to 80% of patients with PM originating from CRC who were treated with CRS and HIPEC are likely to experience recurrence [13, 14], and 10–20% of patients experience metastases to the liver [15, 16].

Treatments for colorectal liver metastases (CRLM) by multi-modality approaches, such as chemotherapy, surgery, and interventional radiology, have recently developed, and surgical resection may result in a 5-year survival rate of 35–45% [17, 18]. However, treatment strategies for metachronous liver metastases following CRS and HIPEC for PM have not been established and little is known about the safety and feasibility of hepatectomy following CRS and HIPEC for PM.

In this report, we describe three cases of metachronous liver metastasis of CRC treated by hepatectomy after CRS and HIPEC for PM.

## Case presentations

### Perioperative management

**Fig. 1:**
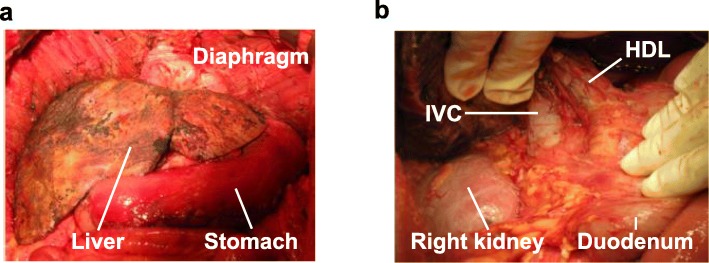
Representative images of the CRS procedure around the liver. **a** The peritoneum of the bilateral diaphragm was stripped, and the liver surface serosa was ablated. **b** Peritonectomy of the hepatoduodenal ligament and retroperitoneum around the liver was performed. The lesser omentum was resected. HDL, hepatoduodenal ligament; IVC, inferior vena cava; CRS, cytoreductive surgery

CRS and HIPEC were indicated for PM from CRC without any other distant metastases. After diagnosis of PM, exploratory laparoscopy was performed, and the size and distribution of the PM was confirmed. If PM was found on the small bowel mesentery, CRS and HIPEC were not indicated. Following neoadjuvant systemic chemotherapy (FOLFOX + cetuximab or bevacizumab) and intraabdominal chemotherapy (paclitaxel) for four cycles, CRS and HIPEC were performed. In CRS, the diseased peritoneum was stripped and organs with PM were resected. Specifically, the CRS procedures around the liver included peritonectomy of the bilateral diaphragm and hepatoduodenal ligament, lesser omentum resection, and ablation of the liver surface serosa (Fig. 1). The peritoneal cancer index (PCI) was used to assess the extent of peritoneal cancer throughout the peritoneal cavity [19]. The presence of residual disease was recorded using the completeness of cytoreduction (CC) score: CC-0, no residual tumor; CC-1, no residual tumor greater than 2.5 mm; CC-2, no residual tumor greater than 25 mm; and CC-3, residual tumor greater than 25 mm [20].

In HIPEC, oxaliplatin (300 mg/m^2^) with 3 L of normal saline was heated to 42 °C and perfused in the abdomen for an hour using a circulating pump. After surgery, 5-fluorouracil (FU) (300 mg/m^2^) was administered intraabdominally through the abdominal drain tube from postoperative days (POD) 1 through 4 as early postoperative intraperitoneal chemotherapy to patients without contraindications, which included bleeding or abdominal distension. Adjuvant chemotherapy was not routinely performed after CRS and HIPEC.

The diagnosis of liver metastasis was performed by computed tomography or magnetic resonance imaging every 6 months after CRS and HIPEC. The indications for hepatectomy included technically resectable liver metastases that preserved a sufficient future liver remnant volume. There was no limit regarding the number or size of tumors when R0 resection was expected to be feasible. The characteristics of the following three cases are shown in Table 1.

**Table 1:** Summary of the cases

	Case 1	Case 2	Case3
Site of the primary tumor	Ascending colon	Cecum	Sigmoid colon
Time from the primary tumor resection to peritoneal metastases (month)	26	12	Simultaneous
Time from the primary tumor resection to CRS and HIPEC (month)	32	17	13
Adjuvant chemotherapy after CRS and HIPEC	None	None	5-FU + LV + bevacizumab 8 cycles
Time from CRS + HIPEC to liver metastases (month)	15	5	6
Chemotherapy before hepatectomy	IRIS + bevacizumab 12 cycles	FOLFOX + bevacizumab 5 cycles	–
Time from CRS + HIPEC to hepatectomy (month)	49	9	6
Operative procedure of hepatectomy	Left hemihepatectomy Partial resection of S5	Posterior sectionectomy	Left lateral sectionectomy Partial resection of S5 and S8
Operative time (min)	269	150	335
Blood loss (ml)	530	233	250
Complications after hepatectomy	Chylous ascites	None	None
Clinical outcome	No recurrence	No recurrence	Death in 16 months after hepatectomy

*Abbreviations*: *CRC* colorectal cancer, *CRS* cytoreductive surgery, *HIPEC* hyperthermic intraperitoneal chemotherapy, *S* segment

### Case 1

**Fig. 2:**
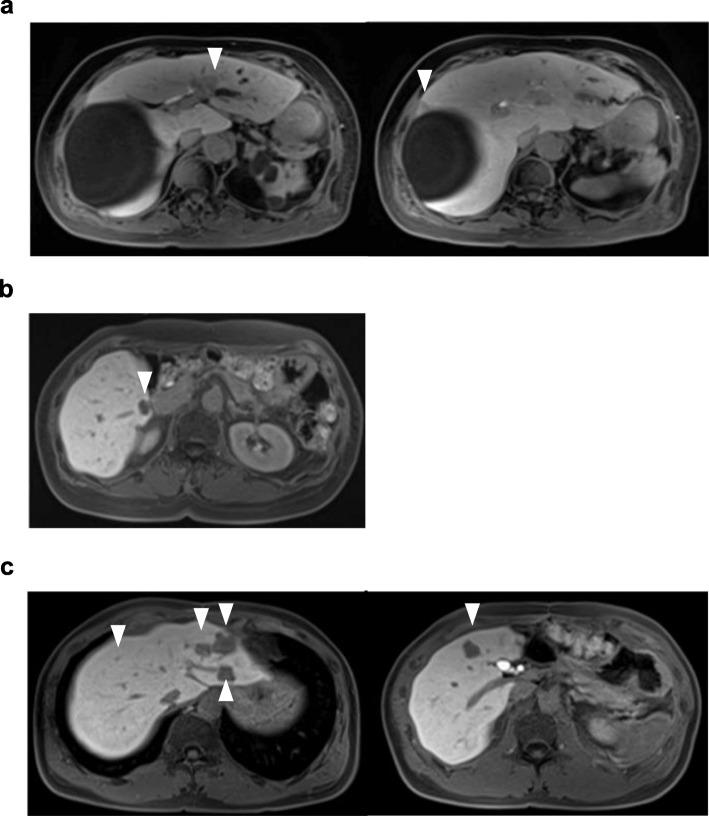
The locations of liver metastases on MRI in the hepatocyte phase. The locations of the tumors are shown with the arrowhead. **a** Case 1. Liver metastases were detected in segments 2 and 5. **b** Case 2. Liver metastases were detected in segment 6. **c** Case 3. Liver metastases were detected in segments 2, 3, 5, and 8. MRI, magnetic resonance imaging

**Fig. 3:**
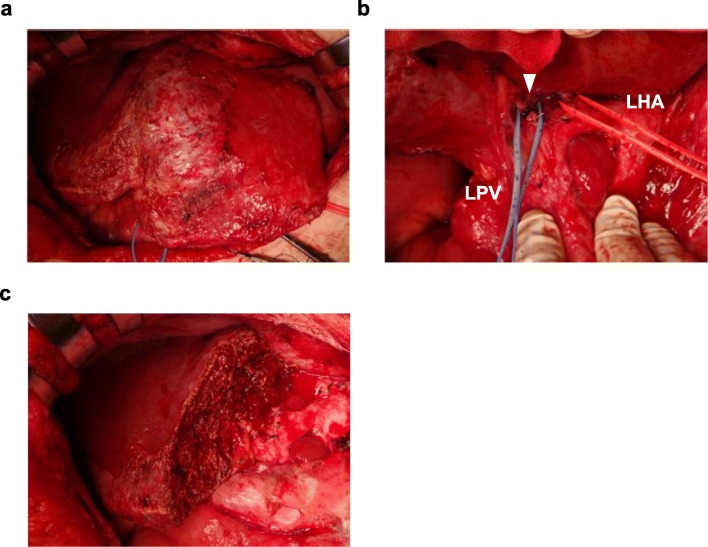
Intraoperative photograph of case 1, where left hemihepatectomy was performed. **a** Adhesion of the liver surface was broad. **b** Dissection of the hepatoduodenal ligament. The left portal vein and left hepatic artery were taped. The middle hepatic artery was cut (arrowhead). **c** Transection surface of the liver. LHA, left hepatic artery; LPV, left portal vein

A 78-year-old woman underwent curative open right hemicolectomy for ascending colon cancer. Pathological diagnosis was well-differentiated tubular adenocarcinoma with K-RAS mutation, T4a, N0, stage IIB [21]. Twenty-six months after the operation, PM was detected and CRS (CC-0) and HIPEC were performed 6 months after adjuvant chemotherapy. Her PCI score was 17/39. After surgery, she developed a surgical site infection and wound dehiscence. She was followed-up without adjuvant chemotherapy. Fifteen months after CRS and HIPEC, liver metastases to segments 2 and 5 were detected (Fig. 2) and systemic chemotherapy (IRIS + bevacizumab, 12 cycles) was initiated because the patient refused surgical treatment. Systemic chemotherapy was continued for 12 months, until discontinuation due to malaise and dizziness. Twenty-two months later, the liver tumor increased in size and dilatation of the peripheral bile duct of the tumor in segment 2 was observed. The patient accepted surgical treatment at that time, and she underwent left hemihepatectomy and partial resection of liver segment 5. Operative time was 4 h and 29 min, and her total blood loss was 530 mL. Broadwide adhesion around the liver was identified and we carefully dissected adhering organs, which included the diaphragm, stomach, duodenum, jejunum, and colon (Fig. 3). It took 2 h and 34 min from the time of the skin incision to the initiation of liver transection. A small amount of chylous ascites were found in the abdomen during the surgery. The postoperative course was uneventful, except for the chylous ascites from the abdominal drain, which gradually subsided after implementation of a fat-restricted diet and diuretics. No recurrence was detected in the absence of adjuvant chemotherapy for 12 months after hepatectomy.

### Case 2

A 57-year-old woman underwent curative laparoscopic ileocecal resection for cecum cancer. Pathological diagnosis was moderately differentiated tubular adenocarcinoma without K-RAS mutation, T3 N1, stage IIIB [21]. Twelve months after the operation, PM and bilateral ovarian metastases were detected and CRS (CC-0) and HIPEC were performed 5 months after adjuvant chemotherapy. The PCI score was 5/39. The postoperative course was uneventful, and she was followed-up without adjuvant chemotherapy. Five months after CRS and HIPEC, liver metastasis to segment 6 was detected (Fig. 2) and systemic chemotherapy (FOLFOX + bevacizumab) was performed. After 5 cycles of chemotherapy over a period of 4 months, the size of the liver metastasis had decreased and no other metastasis or dissemination was detected. Surgical treatment was indicated and posterior sectionectomy of the liver [22] was performed. Operative time was 2 h and 30 min, and her total blood loss was 233 mL. Adhesion of the liver surface to the diaphragm and stomach was found, although it was relatively loose. It took 56 min from the time of the skin incision to the initiation of liver transection. The postoperative course was uneventful, and no recurrence was detected in the absence of adjuvant chemotherapy for 5 months after hepatectomy.

### Case 3

A 38-year-old man underwent Hartmann’s operation for the perforation of the sigmoid colon. Sigmoid colon cancer with PM was detected, and bilateral and diffuse CRLM were also diagnosed during the operation. Pathological diagnosis was moderately differentiated tubular adenocarcinoma without K-RAS mutation, T4a N2, M1c, stage IVC [21]. FOLFOX + bevacizumab was performed for 10 cycles and discontinued because of drug-induced pneumonitis. After the second-line chemotherapy using 5-FU + leucovorin (LV) was performed for 10 cycles, complete remission of liver metastases was achieved. CRS (CC-0) and HIPEC were performed 13 months after the primary tumor resection. The PCI score was 2/39. The postoperative course was uneventful. Adjuvant chemotherapy using 5-FU + LV + bevacizumab was performed because the risk of recurrence was high. After 8 cycles of adjuvant chemotherapy, recurrence of liver metastases in segments 2, 3, 5, and 8 was newly detected (Fig. 2). Six months after CRS and HIPEC, he underwent left lateral liver sectionectomy [22] and partial resection of liver segments 5 and 8. Operative time was 5 h and 35 min, and his total blood loss was 250 mL. The surface of the liver tightly adhered to the abdominal wall, diaphragm, stomach, duodenum, and colon, and we dissected them carefully without damage to other organs. It took 1 h and 37 min from the time of skin incision to the initiation of liver transection. Two months after the hepatectomy, metastases to the liver and lymph nodes were detected. Systemic chemotherapy was performed, but the cancer progressed gradually. The patient died 16 months after hepatectomy.

**Fig. 4:**
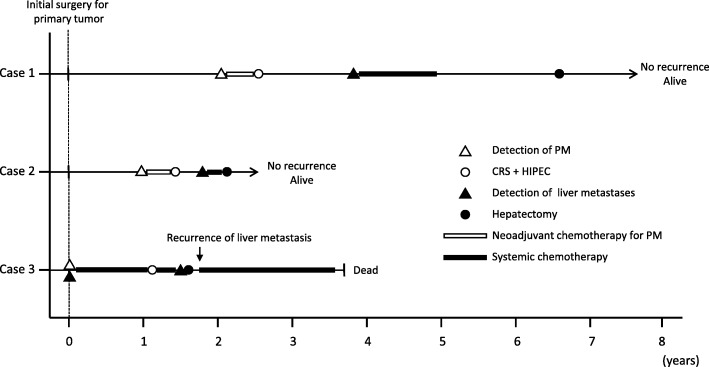
Summary of the clinical courses of the three cases. CRS, cytoreductive surgery; HIPEC, hyperthermic intraperitoneal chemotherapy; PM, peritoneal metastases

The clinical courses of the three cases are summarized in Fig. 4.

## Discussion

CRS and HIPEC were first described in 1980 [23]. This procedure involves stripping of the diseased peritoneum (peritonectomy) with multiple visceral resections and is performed with the goal of achieving a maximal cytoreduction of all visible peritoneal lesions within the abdomen. Following the resections, a heated chemotherapy perfusate is administered intraoperatively into the abdomen to chemically sterilize all peritoneal surfaces. HIPEC allows a high local concentration of a cytotoxic drug to be achieved for microscopic cytoreduction of any residual tumor with minimal systemic adverse effects. In addition, hyperthermia has been demonstrated to have a synergistic effect with chemotherapy, thus enhancing the cytotoxicity of the drug [24].

In the early 1990s, CRS and HIPEC were introduced for CRC patients with PM [6, 25], and these methods have been reported to improve prognosis with 5-year overall survival rates ranging from 27 to 51% [7–12]. With improved prognosis, recurrence after initial CRS and HIPEC has recently been a topic of discussion [16]. Most cases of recurrence have been reported to be intra-abdominal, with the liver being the second most frequent site of recurrence after the peritoneum. About 10–20% of patients develop liver metastases after CRS and HIPEC [15, 16]. Although recent reports suggested that simultaneous hepatectomy for liver metastases with CRS and HIPEC could improve prognosis [26, 27], metachronous hepatectomy after CRS and HIPEC has been scarcely reported. Sánchez-Velázquez et al. reported a case of caudate lobectomy with inferior vena cava resection for liver metastasis from CRC following CRS and HIPEC for colorectal PM, and the patient showed no recurrence for 18 months after hepatectomy [28]. In the present study, we presented three cases of metachronous liver metastases from CRC treated by hepatectomy after CRS and HIPEC for PM. All hepatectomies were successfully performed, and no recurrence was detected in cases 1 and 2, although the observation periods were short. In case 3, liver metastases were diagnosed first; although complete remission was achieved once with systemic chemotherapy, early recurrence was found after hepatectomy for liver metastases. Case 3 showed synchronous presentation of hepatic metastases with a primary tumor, which was reported to be a risk factor for recurrence after CRLM resection, implying that the indication for CRLM resection after CRS and HIPEC should be carefully considered when making a decision about the procedure. However, recent advancement of multi-modality treatment for CRC revealed the efficacy of surgical resection even for metastatic lesions including liver, lung, and peritoneum [29, 30]. Considering these previous reports, surgical approach for liver metastasis after CRS + HIPEC might be justified for better prognosis, although curative and safe resection should be secured.

CRS procedures around the liver include peritonectomy of the bilateral diaphragm and hepatoduodenal ligament, lesser omentum resection, and ablation of the liver surface serosa; therefore, severe adhesion around the liver is expected during reoperation after CRS. In addition, intraabdominal chemotherapy, including HIPEC and early postoperative intraperitoneal chemotherapy, could enhance postoperative adhesion [24]. In the present three cases, hepatectomy was performed after prior CRS and HIPEC. Adhesion of surrounding organs to the liver surface was broadly observed because the peritoneum around the liver was completely stripped in the previous CRS. However, the adhesions could be dissected as in other abdominal surgeries in which the removal of the peritoneum was not aimed, and hepatectomy could be performed safely. This was probably because of the characteristics of peritoneal healing following a peritoneal injury. Fibrinolytic activity over the peritoneal surface is reported to decrease after damage to the peritoneum, leading to changes in the expression and synthesis of various cellular mediators and in the remodeling of connective tissues [31]. Thus, for patients with good performance status and with the ability to combat adverse events, hepatectomy for recurrences in the liver, even after previous CRS and HIPEC procedures, may be associated with long overall survival. However, owing to the shortness of the observational period in the present cases, further studies are needed to show the long-term outcomes of hepatectomy for liver recurrences after previous CRS and HIPEC. In addition, the optimal timing of hepatectomy for liver metastasis after CRS + HIPEC including the interval between liver metastasis and CRS + HIPEC and conversion from systemic chemotherapy should be investigated for more efficacious multi-modality treatment to advanced CRC.

## Conclusion

In conclusion, three cases of hepatectomy for CRLM after CRS and HIPEC were reported in the present study. Although adhesion around the liver was severe and re-laparotomy could have been difficult, the adhesions could be dissected safely. Hepatectomy for metachronous liver metastasis after CRS and HIPEC was feasible in specialized centers and could be considered as a multi-modality treatment option for advanced CRC recurrence.

## Data Availability

The datasets used and/or analyzed during the current study are available from the corresponding author on reasonable request.

## Abbreviations

CC: Completeness of cytoreduction
CRC: Colorectal cancer
CRLM: Colorectal liver metastases
CRS: Cytoreductive surgery
FU: Fluorouracil
HIPEC: Hyperthermic intraperitoneal chemotherapy
LV: Leucovorin
PCI: Peritoneal cancer index
PM: Peritoneal metastases
